# Plan Beta for tuberculosis: it's time to think seriously about poorly ventilated congregate settings

**DOI:** 10.5588/ijtld.15.0494

**Published:** 2016-01

**Authors:** T. A. Yates, F. Tanser, I. Abubakar

**Affiliations:** *Centre for Infectious Disease Epidemiology, Research Department of Infection and Population Health, University College London, London, UK; †Wellcome Trust Africa Centre for Population Health, University of KwaZulu-Natal, Mtubatuba; ‡School of Nursing and Public Health, University of KwaZulu-Natal, Durban, South Africa; §Medical Research Council Clinical Trials Unit, University College London, London, UK

**Keywords:** tuberculosis, epidemiology, infection control

## Abstract

Globally, the rates of decline in tuberculosis (TB) incidence are disappointing, but in line with model predictions regarding the likely impact of the DOTS strategy. Here, we review evidence from basic epidemiology, molecular epidemiology and modelling, all of which suggest that, in high-burden settings, the majority of Mycobacterium tuberculosis transmission may occur in indoor congregate settings. We argue that mass environmental modifications in these places might have a significant impact on TB control and suggest a research agenda that might inform interventions of this nature. The necessary technology exists and, critically, implementation would not be dependent on health care workers who are in short supply in the communities worst affected by TB.

## RESUME

Le déclin de l'incidence de la tuberculose (TB) dans le monde est décevant mais en accord avec les prédictions modélisées relativesà l'impact probable de la stratégie DOTS. Nous revoyons ici les preuvesémanant de l'épidémiologie de base, de l'épidémiologie moléculaire et de la modélisation qui suggèrent toutes que, dans un contexte durement frappé, la majorité de la transmission de Mycobacterium tuberculosis survient dans des lieux fermés de rassemblement de population. Nous argumentons en faveur de modifications de masse de l'environnement dans ces endroits qui pourraient avoir un impact significatif sur la lutte contre la TB et suggérons un programme de recherche qui pourrait renseigner des interventions de cette nature. Les techniques nécessaires existent et, chose cruciale, leur mise en œuvre ne serait pas dépendante des travailleurs de santé qui sont en nombre réduit dans les communautés les plus affectées par la TB.

## RESUMEN

En general, las disminuciones en la incidencia de la tuberculosis (TB) son desalentadoras, pero corresponden a las predicciones de los modelos sobre la posible repercusión de la estrategia DOTS. En el presente artículo se examinan los datos existentes en materia de epidemiología fundamental, epidemiología molecular y de estudios de modelización y todos indican que en los entornos con una alta carga de morbilidad, la mayor parte de la transmisión de Mycobacterium tuberculosis ocurre en ambientes colectivos interiores. Se trata de demostrar que las modificaciones ambientales en los entornos colectivos podrían tener una repercusión considerable en el control de la TB y se propone un programa de investigación que podría fundamentar las intervenciones de este tipo. La tecnología necesaria existe y su aplicación no estaría a cargo de los profesionales de salud, cuya escasez es notoria en las comunidades más afectadas por la TB.

REDUCTIONS IN TUBERCULOSIS (TB) incidence are particularly disappointing in the high-burden Southern African nations with generalised human immunodeficiency virus (HIV) epidemics that are the focus of this article. In these communities, very high rates of ongoing Mycobacterium tuberculosis transmission have been observed. In places, the annual risk of tuberculous infection exceeds 5%.^1^,^2^

## THE MODE OF TRANSMISSION OF M. TUBERCULOSIS

Early studies in the Johns Hopkins Experimental TB Ward demonstrated that TB was spread via respiratory droplets.^3^ Wells calculated that, while larger respiratory droplets would rapidly fall to the floor, smaller droplets, with high surface area to volume ratios, would rapidly evaporate, leaving ‘droplet nuclei’.^4^ These tiny particles would remain suspended on currents of air until either inhaled or ventilated out of the room. To cause disease, M. tuberculosis needs to avoid filtration in the nose and respiratory tract and to come into contact with an alveolar macrophage. Both Wells et al.^5^ and Lurie et al.^6^ demonstrated that it was hard to induce infection by exposing rabbits to large droplets, whereas a lower dose of M. tuberculosis in smaller droplets reliably produced infection. Recent cough box experiments in Uganda have shown that, among droplets produced directly by coughing, most culturable M. tuberculosis was found in the smallest droplets,^7^ and production of such droplets predicted transmission to household contacts.^8^

Given that, in poorly ventilated spaces, droplet nuclei might remain suspended in the air for prolonged periods, it seems plausible that crowd contact (time in the same enclosed space as an infectious individual, either at home or in a congregate setting) might play a more important role than other forms of contact (for example, conversational contact) in M. tuberculosis transmission. This assertion is supported by well-described TB outbreaks.^9^

## HIGH RATES OF INFECTION AND DISEASE ASSOCIATED WITH USE OF PUBLIC SPACES

Individuals with greater exposure to indoor congregate settings have a greater risk of tuberculous infection and TB disease. For example, regular use of public transportation has been linked to infection and disease in Lima, Peru,^10^–^13^ and to TB disease in Houston, TX, USA.^14^ While there were methodological problems with some of these studies, the consistency of the findings, biological plausibility and strong effect estimates suggest a causal relationship between exposure to public transportation and TB.

Such studies raise the intriguing possibility that the elevated risk of TB associated with working in health care facilities, prisons or mines might be, at least in part, simply a consequence of exposure to a poorly ventilated indoor public space. After adjustment for factors including HIV and silicosis grade, miners working below ground have twice the TB incidence rate of miners working on the surface.^15^

The association between household contact and M. tuberculosis infection wanes with increasing TB burden,^16^ suggesting, as might be expected, that transmission in indoor congregate settings is particularly important in high-burden settings.

## MOLECULAR EPIDEMIOLOGY SUGGESTS LIMITED INTRA-HOUSEHOLD TRANSMISSION

A small number of studies from Southern Africa have attempted to concurrently type strains and obtain data on household residence from all individuals with TB within defined communities.^17^–^20^ Such studies can estimate the proportion of TB disease that results from recent transmission between household members. Strong assumptions are required, including that two co-prevalent cases of TB in the same home and with the same strain type means recent transmission within the home. Estimates might be biased in either direction. For example, if two residents in the same home were both infected by a third individual, perhaps at church, the proportion of transmission in households would be overestimated. However, if there were a limited number of circulating strains or the strain typing technique had limited resolution, reactivation cases might wrongly be assumed to be due to recent infection. Given that most cases of TB are in households without other cases,^21^ the relative contribution of transmission in indoor congregate settings might be overestimated. All such molecular epidemiology studies in Southern Africa have suggested that transmission within households makes a modest contribution to transmission (Table).^17^–^20^ It should be noted that the methods used to derive these estimates differed, and that the studies from Malawi included non-resident relatives.^18^,^20^

**Table:**
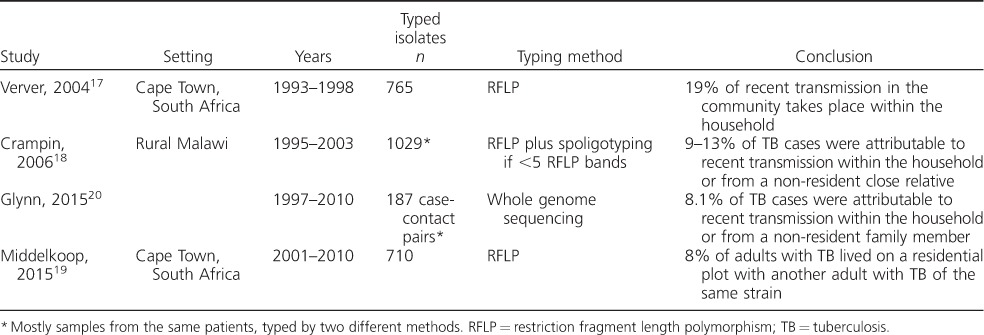
Molecular epidemiology studies from Southern Africa estimating the proportion of M. tuberculosis transmission occurring within households

This should, perhaps, come as little surprise given the limited yield of household case finding,^21^ and given that most disease in high-burden settings is a result of recent transmission.^22^ These proportions should be interpreted as reflecting a high risk of transmission outside the home rather than any attenuation in the risk of household transmission. In support of this position, a small molecular epidemiology study from Uganda suggested that transmission can only be understood if both household contacts and crowd contacts in public spaces are considered.^23^

## MODELLING TRANSMISSION USING THE WELLS-RILEY EQUATION

According to the Wells-Riley Equation and its variants,^24^ the risk of transmission of respiratory infections via crowd contact is a function of time spent in a defined space, the number of infectious individuals present, how many ‘infectious quanta’ they produce per unit time (usually fitted or assumed), the rate at which susceptible individuals breathe, and the rate at which the room is ventilated with ‘germ-free air’. Combining this approach with data on social contact patterns, a study from Cape Town, South Africa, suggested that in that setting 84% of all M. tuberculosis transmission occurs outside one's own household, largely in schools, workplaces and on public transport.^25^ However, in that study, it is unclear how the timing of indoor carbon dioxide (CO_2_) measurements related to building occupancy.^25^ Furthermore, as marked spatial and temporal variations in outdoor CO_2_ levels should be expected, the lack of contemporaneous local outdoor CO_2_ measurements is a limitation. This may have affected the precision of the estimates.

There are, in addition, inherent limitations to the Wells-Riley approach, including strong assumptions about air mixing, that location-specific data on TB prevalence are not usually available, and that, as discussed below, it does not account for heterogeneity in infectiousness or susceptibility.

## HETEROGENEITY

Substantial heterogeneity in the infectiousness of people with TB has been demonstrated by a range of methods.^8^,^26^–^29^ While some of this heterogeneity might be within patients over time rather than true between-patient heterogeneity, it is certainly true that, at any moment, some patients are much more infectious than others. Let us suppose that there is a small number of very infectious individuals who are the source of most infections. Most individuals would only come into contact with a ‘super-spreader’ in public spaces. We would therefore expect heterogeneity in infectiousness to increase the proportion of transmission occurring in public rather than private spaces.

In Southern Africa, there is also substantial heterogeneity in susceptibility to TB as a result of HIV-related immunocompromise. This additional heterogeneity would, in our view, also increase the proportion of transmission occurring outside the household. The probability of a super-spreader and an individual with a low CD4 count coming into contact would be greater in spaces with more occupants. Furthermore, within a household there is a finite limit on the number of individuals a super-spreader can infect. This is not the case in indoor congregate settings.

Estimates of M. tuberculosis transmission risk made using the Wells-Riley approach do not account for this heterogeneity. The suggestion that, in Cape Town, 84% of M. tuberculosis transmission occurs outside one's own household might therefore be an underestimate.^25^

## PLAN BETA

The effective reproductive number of a communicable disease approximates the product of the number of contacts individuals make per unit time, the duration of infectiousness, the proportion of the population that is susceptible to the pathogen in question and the effective contact rate. The effective contact rate, commonly denoted as ‘beta’, is the average risk of transmission per contact between an infectious and a susceptible individual.

TB control programmes currently focus on reducing the duration of infectiousness through case detection and treatment completion. Might TB control be substantially improved if other components of the effective reproductive number were also targeted? Modelling suggests that TB incidence is very sensitive to changes in beta,^30^ defined here as ‘the number of secondary TB infections per smear-positive person-year’. Reductions in the number of contacts would be hard to achieve, but TB control strategies could attempt to make crowd contact less ‘effective’.

If, in high-burden settings, M. tuberculosis transmission occurs primarily in indoor congregate settings, targeted infection control interventions in these spaces may be a good idea. This article focuses on mass environmental modifications, which we believe might be an effective TB control strategy with several attractive features (see below). This approach should be complemented, where feasible, by other components of TB infection control: administrative controls and the appropriate use of personal protective equipment. A thoughtful article on administrative controls was recently published in this *Journal*,^31^ and we await the results of the F-A-S-T trial with interest.^32^

## KNOWN UNKNOWNS: A RESEARCH AGENDA

If we are to take this idea forward, the following critical questions should be addressed.

### Where precisely does M. tuberculosis transmission occur in high-burden settings?

Early molecular epidemiology suggested that 70% of non-reactivation TB in Los Angeles, CA, USA, was due to transmission in three homeless shelters.^33^ Similarly detailed insights are yet to be made in high-burden settings, but would be of huge value in designing control programmes. A number of settings—mines, prisons and health care facilities—are clearly important sites of TB transmission in Southern Africa, but it is unclear what proportion of TB transmission they can explain. The evidence summarised above suggests that transmission between members of the same households makes only a modest contribution to M. tuberculosis transmission in high-burden settings. However, these data do not exclude the possibility that much transmission might occur when people visit each other's homes, i.e., where private space is used as public space.

Upper room ultraviolet germicidal irradiation (UVGI) can reduce TB transmission by 70%.^34^ High rates of natural ventilation may be even more effective.^35^ If it is true that most transmission in high-burden settings occurs in a limited number of indoor public spaces, retrofitting buildings to improve ventilation and or installing UVGI might be effective interventions. However, if transmission occurs in a wider range of settings, including households, regulation and proactive involvement in large building projects, such as South Africa's Reconstruction and Development Programme, may be better strategies.

There have been, to our knowledge, no attempts to associate exposure to putative sites of transmission in Southern Africa with measured M. tuberculosis infection. We are currently undertaking such a study in KwaZulu-Natal. Our study is cross-sectional in nature, using Mantoux tests in children. However, in high-burden settings, TB infection incidence cohorts may be feasible, allowing investigators to be more confident that data collected on use of public spaces approximate exposure at the time of infection. Molecular approaches could also be used to compare the social contact patterns of individuals with TB disease resulting from recent infection to controls. Regardless, quality data should be collected on exposure to putative sites of M. tuberculosis transmission using, for example, occupational cohorts or detailed social contact pattern diaries. Developments in our ability to measure both infection and use of indoor public space may help.

The validation of an M. tuberculosis-specific skin test is keenly awaited.^36^ Other gaps in our armoury include tools to measure reinfection, which is common in high-burden settings,^1^ and to reliably measure tuberculous infection in HIV-positive people. Social contact patterns are difficult to measure. Global positioning system (GPS) devices allow detailed measurement of the indoor spaces to which individuals are exposed.^37^ Coupling this technology with measurements of incident infection could be powerful.

A radical alternative approach would be to attempt to quantify aerosolised M. tuberculosis directly in the putative sites of transmission. Polymerase chain reaction of room air filtrate has been piloted successfully in clinical environments.^38^ Comparing quantitative measures of aerosolised M. tuberculosis at sites known to carry a transmission risk and at proposed sites of transmission would be informative.

### Are improvements in ventilation in these spaces feasible, acceptable and affordable?

The involvement of experts in big building projects and in hospital and prison retrofits seems prudent. However, a set of standardised building designs and retrofits that can be implemented by contractors will be needed if environmental modifications are to be implemented more widely. Environmental improvements in some settings may be limited by factors such as climate and availability of electricity. They may not be acceptable to communities or be prohibitively expensive. Discussions with affected communities and costed pilot interventions will be needed to inform the development of protocols and to quantify expected uptake and the reduction in transmission risk that might be achieved.

### How big are people's networks of shared indoor air?

Community randomised trials of interventions to interrupt transmission will eventually be needed (see below). To make these efficient and avoid contamination—the intervention altering TB rates in control clusters—we need a better understanding of the scale of M. tuberculosis transmission networks. This could be gained through detailed qualitative research or using the GPS technologies described above.

## THE ‘SLEDGEHAMMER TRIAL’

A community-randomised trial in which, in intervention communities, putative sites of M. tuberculosis transmission are made safe through environmental modifications has been labelled the ‘sledgehammer trial’. Fortunately, breaking windows is not the only means of improving ventilation.^35^ The Tuberculosis Ultraviolet Shelter Study was a community-randomised trial of upper room UVGI conducted in American homeless shelters.^39^ While there were too few incident tuberculous infections to demonstrate an impact on transmission, the study showed that mass environmental modification was feasible under trial conditions. In high-burden settings, the event rate would be much higher.

A community-randomised trial of mass environmental modifications in putative sites of M. tuberculosis transmission could answer many of the questions posed above. The outcome could be incident tuberculous infections in a cohort of non-infected individuals recruited at baseline or be measured through repeated tuberculin surveys. While better observational data might make the intervention more efficient by, for example, not intervening at sites where limited transmission occurs, this will likely differ by community. Moreover, estimates derived from observational studies may prove inaccurate given the challenges inherent both in measuring social contact patterns and in measuring TB transmission in populations. Given the potential advantages of the strategy, we feel the trial should not be unduly delayed if data from observational studies are not forthcoming. In our view, such a trial would be ethical given that large-scale environmental modifications are not being undertaken currently, the putative harms of the intervention (e.g., loss of thermal comfort, loss of privacy or increased risk of break-ins), and the feasibility of implementing the intervention at scale if the trial showed significant benefits.

Transmission models incorporating data on the use of public spaces, the acceptability of environmental modifications and the rates of ventilation (or disinfection) that can be achieved could be used to estimate the necessary sample size and study duration.

## POTENTIAL ADVANTAGES OF ENVIRONMENTAL MODIFICATION AS A STRATEGY

In settings with well-functioning DOTS-based programmes, incremental improvements in case finding and treatment completion may become increasingly difficult. Synergism might be expected between improved case finding and environmental modification, with missed cases less able to transmit to others while still alive and untreated. Other advantages include the generation of employment in the construction industry; potential reductions in the transmission of other respiratory infections and in non-communicable diseases caused by passive smoking or indoor air pollution; sustainability, given that many retrofits would not need any further work; and that the technology is both cheap and already available. In settings with social gradients in access to health care, the approach may also be more equitable. However, given the critical personnel shortages in most high-burden countries, the most important advantage of this approach is that it does not place additional demands on health care workers.

## BACK TO THE FUTURE

The decades before effective biomedical interventions against TB became available saw steep falls in TB deaths across the industrialised world. Modelling suggests that, from at least 1900, declines in TB burden in England and Wales were largely a result of reductions in effective contacts made by each infectious case rather than reductions in the rate of progression from infection to disease.^40^ The authors note that this does not exclude a role for nutrition, as better-nourished individuals may not become infected given the same exposure. So-called ‘early clearance’ is poorly understood. However, an examination of TB mortality in earlier decades showed little link between food availability and TB.^41^ Newsholme described strong temporal associations in a number of settings between declines in TB mortality and isolation of infectious TB patients both formally, in sanatoria, and de facto isolation in workhouses as a result of England's Poor Laws.^42^

Today, treatment rather than isolation is our primary means of reducing the duration of infectiousness. The DOTS approach has undoubtedly saved many lives, but, in line with model predictions, has resulted in only modest falls in incidence.^30^ New vaccines are years away and interventions to alter contact patterns are probably not realistic. In the medium term, ‘sledgehammer’ interventions to reduce beta may be the most feasible means of reducing the effective contact rate, thus augmenting the declines TB incidence achieved via DOTS. Research to inform such a strategy could start immediately and should be prioritised.
